# Reflective grating-coupled structure improves the detection efficiency of THz array detectors

**DOI:** 10.1038/s41598-018-26204-y

**Published:** 2018-05-23

**Authors:** Peng Xiao, Xuecou Tu, Lin Kang, Chengtao Jiang, Shimin Zhai, Zhou Jiang, Danfeng Pan, Jian Chen, Xiaoqing Jia, Peiheng Wu

**Affiliations:** School of Electronic Science and Engineering, Nanjing University, Nanjing, 210023 China

## Abstract

A reflective grating-coupled structure on the silicon substrate was designed to improve the detection efficiency of terahertz detectors for the frequency ranging from 0.26 THz to 0.36 THz. By using finite difference time domain (FDTD) solutions, the simulation and optimized design of the grating-coupled structure were carried out. The results showed that the signal was effectively reflected and diffracted by the reflective grating-coupled structure which significantly enhanced the electric field in the place of the detector. The maximum electric field can be increased by 2.8 times than that of the Fabry-Perot resonator. To verify the design results, the reflective grating-coupled structure was applied in the preparation of the Nb_5_N_6_ array detector chip and compared with the Nb_5_N_6_ array detector chip with the F-P resonator. The results showed that the maximum voltage responsivity of the Nb_5_N_6_ detector with the reflective grating-coupled structure was 2 times larger than the Nb_5_N_6_ detector with the F-P resonator. It indicates that the reflective grating-coupled structure can efficiently improve the detection efficiency of THz detectors.

## Introduction

Terahertz (THz) detectors, which has attracted great attention and been applied in many fields such as security check, medical imaging and components identification^1,2^, actually play a very important role in the field of the THz (0.1 THz~10 THz) technology. Therefore, it is of great significance to further improve the detection efficiency and sensitivity of the THz detector^3–6^. In the THz frequency range, the Gaussian beam is usually used to describe the transmission characteristics of the THz wave. Subject to the diffraction limit, the lower bound of the Gaussian beam waist is about 0.9003λ in the THz quasi-optical system^7^. That being said, for THz detector prepared by microfabrication, the size of the focused Gaussian beam waist is much larger than that of the detector. Such limitation leads to a low signal coupling efficiency. Therefore, many coupling structures have been applied to improve the signal coupling efficiency of THz detector, for example, the extended hemispherical lens^8^, diffractive lens^9^, F-P air cavity^10–12^ and metal grating coupler^13,14^, etc. However, the sizes of the extended hemispherical lens and the diffractive lens are much larger than that of the detector. In addition, the preparation processes are complicated and expensive, hence makes it harder to be applied to fabricate large-scale detectors array. The F-P air cavity is used in the infrared band to improve the detection efficiency of the detector, but its effectiveness is limited in THz band. Based in that, some innovative concepts and structures are gradually applied to replace traditional geometric optics in order to eliminate these limitations. For example, flat optics can produce abrupt changes over the scale of the free-space wavelength in the phase, amplitude and polarization of a light beam by assembling arrays of miniature, and anisotropic light scatterers. The size and the period of the unit are much smaller than the wavelength. According to Huygens principle, flat optics can mould optical wavefronts into arbitrary shapes with subwavelength resolution by introducing spatial variations in the optical response of the light scatterers^15^. Therefore, it can create smaller and more optically efficient products, and at the same time, are also gradually used in THz band. The detection efficiency can be improved by using the sub-wavelength periodic structure to regulate the phase and amplitude of the signal^16–20^.

In this paper, a reflective grating-coupled structure with sub-wavelength size and high coupling efficiency was designed and fabricated for Nb_5_N_6_ array detector. The experimental results verified that with the reflective grating-coupled structure, the maximum voltage responsivity of Nb_5_N_6_ array detector was 2 times larger than that of detectors array with a F-P resonator.

## Design and Results

**Figure 1:**
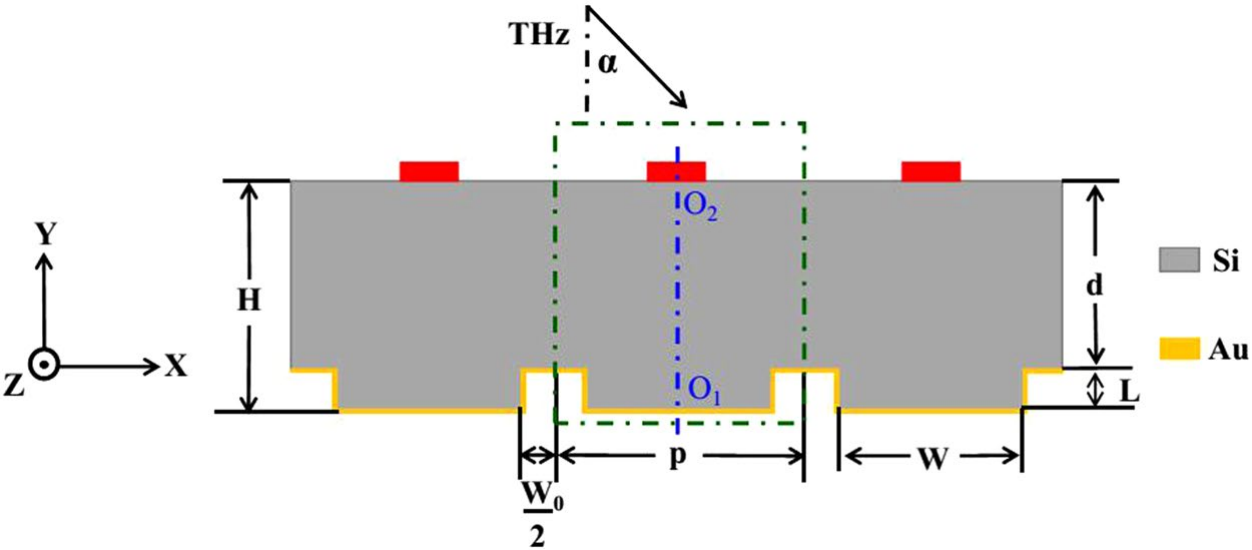
The cross-section of the reflective grating-coupled structure. The red area indicates the place of the detector, the blue dotted line is the centerline of a periodic unit cell (P).

To improve the detection efficiency of the THz array detector, a reflective grating-coupled structure on the basis of the F-P resonator was designed, i.e. a grating structure with a certain width and depth is attached to the back of Si substrate, and it is covered by a 200 nm-thick Au film, as shown in Fig. 1.

Here, *H* is the substrate thickness, *d* is the height of the grating, *L (L* = *H* *− d)* is the height of the grating gear, *W* is the width of the grating gear, *W*_0_ is the width of the groove and *P (P* = *W* + *W*_0_) is the grating period. The red area indicates the place of the THz array detector. The blue dotted line is the centerline of the grating period; the O_1_ and O_2_ are the center point of the grating gear and detector, respectively. For the reflective grating-coupled structure, a portion of the incidence signal is reflected at the substrate surface, and the resident signals transmit into the silicon substrate. The transmission signals at the grating gear place can be totally reflected, which is similar to the case of F-P cavity. Besides, the transmission signals at the groove place can be diffracted due to the grating effect. So the reflected and diffracted signals in substrate could be effectively coupled to the substrate surface, and the signals in the place of the detector can be enhanced if their phases are equivalent. The reflective grating-coupled structure makes the signal in the place of the detector larger compared with the F-P cavity.

The simulation design of the reflective grating-coupled structure is performed by using FDTD software (FDTD Solutions, Lumerical Inc.) in a two-dimensional (2D) system–the X-Y plane in Fig. 1. Because of the periodic condition of the reflective grating-coupled structure, the simulated object is a unit cell (shown as a green dotted rectangle in Fig. 1), which has periodic boundary conditions (PBC) on its left and right boundaries and perfect matched layer (PML) conditions on its top and bottom boundaries. In the simulations, a polarized plane wave with the electric (E) field amplitude of 1 V/m, propagates along the Y-axis, is employed as the incident signal (*α* = 0°). The grid size is set to 1 μm which is much smaller than the unit sizes and the operating wavelength.

**Figure 2:**
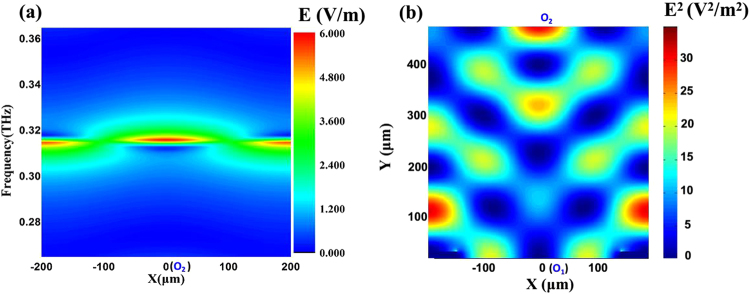
Simulated focusing characteristics, when *H* = 500 μm, *L* = 15 μm, *W*_0_ = 100 μm, and *P* = 400 μm. (**a**) Frequency dependence of the *E* field along the X-axis on the Si substrate surface. (**b**) *E*^2^ distribution on the cross-section of the substrate in a periodic unit cell at 0.318 THz.

According to the simulation and optimized design results, a group of optimized parameters *H* = 500 μm, *L* = 15 μm, *W*_0_ = 100 μm and *P* = 400 μm are selected. Figure 2(a) presents a frequency dependence of the *E* field on Si substrate surface in this case. Within 70 μm radius of substrate surface, the electric field is enhanced significantly at a frequency range from 0.315 THz to 0.32 THz. In other words, the signal is converged on the detector location of Si substrate surface highly effectively by the reflective grating-coupled structure. Figure 2(b) is the *E*^2^ (f = 0.318 THz) distribution on the cross-section of Si substrate in a periodic unit cell, revealing the transformation process from the incident plane wave to the converging wave. It demonstrated the reflective and diffractive effects due to this unique structure, resulting in a focusing electric intense profile.

**Figure 3:**
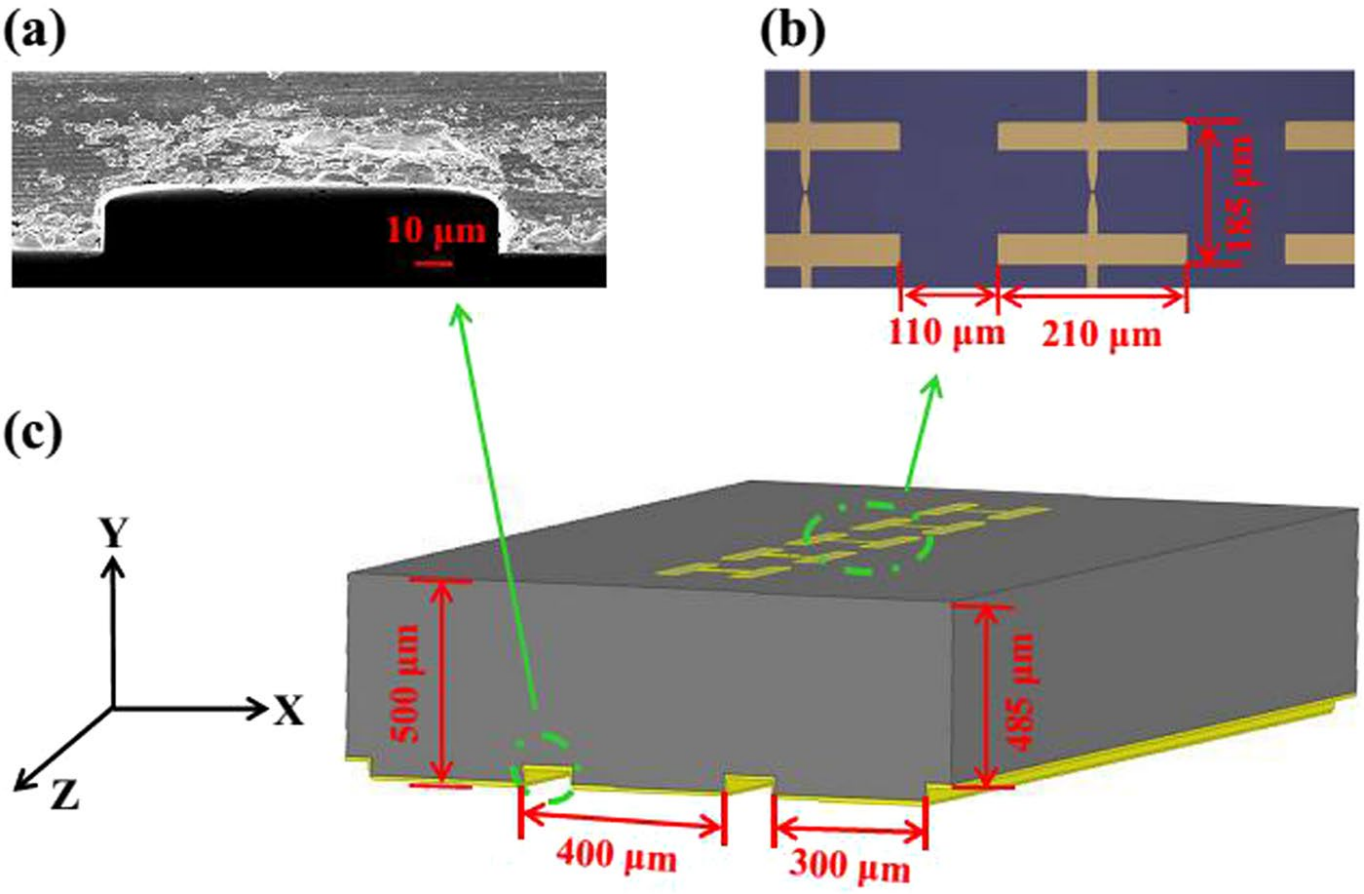
(**a**) A cross-section SEM picture of the grating groove. (**b**) Optical microscope image of Nb_5_N_6_ THz array detectors, the size of a single detector is 210 μm × 185 μm, and the period of the detectors array is 320 μm. (**c**) Schematic of the reflective grating-coupled structure.

To verify the design results, based on the optimal parameters, the reflective grating-coupled structure was applied in the preparation of the Nb_5_N_6_ array detector. Nb_5_N_6_ detector mainly consists of a gold dipole planar antenna and Nb_5_N_6_ thin film microbridge as shown in Fig. 3(b). When the detector is irradiated by THz waves, the signal can be received by the dipole antenna and coupled to the Nb_5_N_6_ microbridge. After receiving the energy from the irradiated power, the resistance of the detector changes with the Joule heating. By applying a bias current to the detector, the change in resistance can be converted to a voltage change.

And the measured results are compared with Nb_5_N_6_ array detector integrated with a F-P resonator, which has a maximum responsivity of 580 V/W^21^. The optimization parameters of the reflective grating-coupled structure are shown in Fig. 3(c). The Si substrate thickness *H* is 500 μm, the grating period *P* is 400 μm, the height of the grating *d* is 485 μm, and the width of grating gear *W* is 300 μm. Each grating periodic unit contains an Nb_5_N_6_ detector at its surface center. Figure 3(a) is a cross-section SEM picture of the grating groove and Fig. 3(b) shows an optical microscope picture of Nb_5_N_6_ array detector chip, respectively.

**Figure 4:**
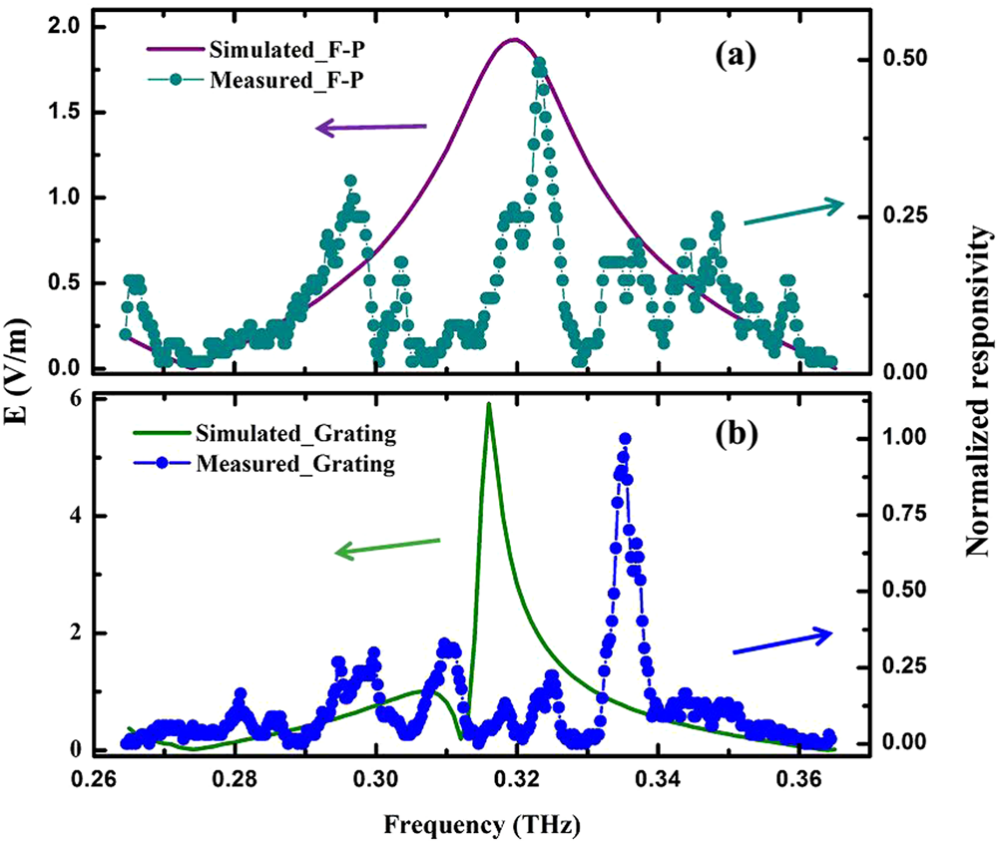
(**a**) The cyan dotted line shows the normalized voltage responsivity of the Nb_5_N_6_ detector with the F-P resonator. The purple solid line shows the simulated value of *E*_*x* = *0, y* = *480*_ on the F-P resonator. (**b**) The blue dotted line shows the normalized voltage responsivity of the Nb_5_N_6_ detector with the reflective grating-coupled structure. The green solid line shows the simulated value of *E*_*x* = *0, y* = *500*_ on the reflective grating-coupled structure.

To facilitate results analysis, the measured voltage responsivity of Nb_5_N_6_ array detector with two structures were normalized and compared with the simulated results. For impedance detector integrated with a F-P resonator, a normalized voltage responsivity is shown in Fig. 4(a). The purple solid line is the relations between simulated electric fields and frequency. A maximum value of 1.94 V/m is at 0.318 THz. Here, a 480 μm-thick silicon wafer is used as the substrate of the F-P resonator to ensure that the resonant frequency lies in the frequency range of 0.315 THz to 0.32 THz. The cyan dotted line is the measured results, with a maximum normalized voltage responsivity of 0.5 at 0.323 THz. For Nb_5_N_6_ detector integrated with a reflective grating-coupled structure, the green solid line in Fig. 4(b) shows the dependence of the simulated electric fields on frequency, a maximum value of 5.6 V/m is at 0.318 THz. The blue dotted line in Fig. 4(b) presents the variation in measured values with the frequency; a maximum normalized voltage responsivity of 1 is obtained at 0.335 THz, which is 2 times higher than that of Nb_5_N_6_ detector with a F-P resonator. In particular, since the incident signal entered substrate is reflected and diffracted by the grating structure; the bandwidth is only 4 GHz, which is smaller than that of the detector with the F-P resonator.

There is a small shift at the resonance frequencies in Fig. 4. Such deviation could be due to the imperfection when matching between the Nb_5_N_6_ microbridge impedance, dipole planar antenna impedance and wave impedance in free space. Thus, this small shift should not be considered as the effect of the size deviation in the fabrication process.

## Discussion

**Figure 5:**
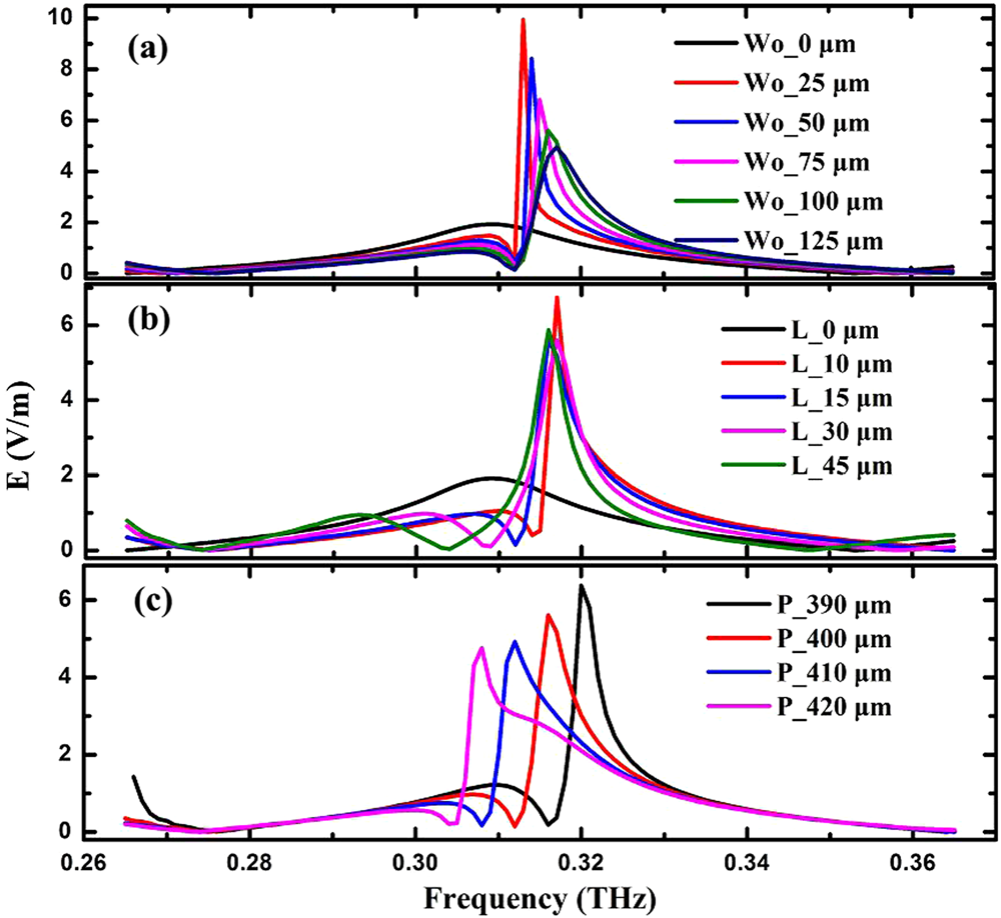
The relations between different frequencies and the *E* field in the place of the detector under different parameters: (**a**) *H* = 500 μm, *L* = 15 μm, *P* = 400 μm, *W*_*0*_ = 0~125 μm; (**b**) *H* = 500 μm, *P* = 400 μm, *W*_*0*_ = 100 μm, *L* = 0~45 μm; (**c**) *H* = 500 μm, *L* = 15 μm, *W*_*0*_ = 100 μm, *P* = 390~420 μm.

To ensure the resonant frequency of the reflective grating-coupled structure is between 0.26 THz to 0.36 THz, *H* = 500 μm is firstly chosen. To make use of the first order diffraction, the grating period should be smaller than the wavelength in free space, but larger than that in silicon^19^. Moreover, 400 μm grating period is chosen based on the detector size, the signal amplitude, the bandwidth and the processing technology. By using FDTD Solutions, the influence of structural parameters on the coupling ability of the reflective grating-coupled structure was investigated. Based on the three parameters, *H* = 500 μm, *P* = 400 μm and *L* = 15 μm, Fig. 5(a) shows the effect of changing *W*_0_ from 0 to 125 μm. It was a F-P resonator when *W*_0_ = 0 μm, associated with a peak value of 1.97 V/m electric field on the surface of Si substrate. When the *W*_0_ was increased to 25 μm, because of the reflective grating-coupled structure, the electric field in the place of the detector was significantly increased to 9.95 V/m (about 5 times comparing to the electric field value of the F-P resonator). But the full width at half maximum (FWHM) of the electric field peak was only 1.25 GHz. Increasing *W*_0_ from 25 μm to 125 μm, the electric field peak gradually decreased with the bandwidth gradually increased. At the value of 125 μm, the electric field peak was reduced to 4.95 V/m, but the FWHM of the electric field peak risen to 8 GHz.

At the value of *W*_0_ = 100 μm, an optimal combination of a relatively high electric field (5.6 V/m) and proper bandwidth (6 GHz) would be achieved. Thus, Fig. 5(b) shows the results using parameters *H* = 500 μm, *P* = 400 μm and *W*_0_ = 100 μm, and changing the height of the grating gear (*L*). The structure was also a F-P resonator when *L* was 0 μm. When *L* was increased to 10 μm, the electric field in the place of the detector was significantly enhanced and the maximum value reached up to 6.6 V/m, the FWHM of the electric field peak was 4 GHz. When *L* was 15 μm, the electric field peak was reduced to 5.6 V/m, but the FWHM of the electric field peak increased to 6 GHz. No significant change was found in field peak and bandwidth if further increasing *L*.

At last, the results of changing the grating period (*P*) are shown in Fig. 5(c), when *H* = 500 μm, *L* = 15 μm and *W*_0_ = 100 μm. Similar as before, *L* = 15 μm was chosen because an relatively optimal combination can be achieved. Increasing *P* from 390 μm to 420 μm, the electric field peak shifted towards the direction of the lower frequency. In other words, the peak values decreased from 6.38 V/m to 4.76 V/m and the resonant frequency changed from 0.32 THz to 0.308 THz, corresponding to *P* from 390 μm to 420 μm.

**Figure 6:**
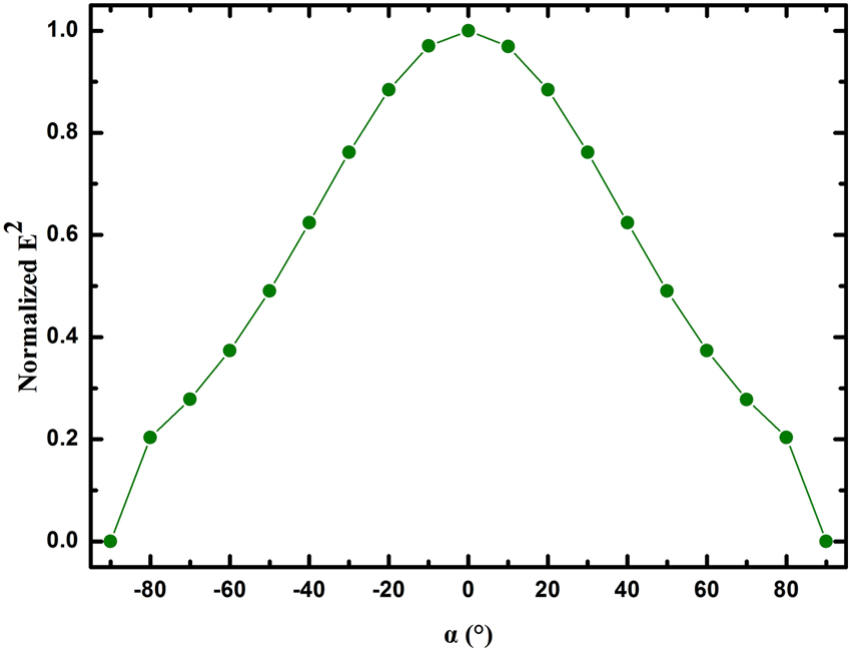
The dependence of normalized *E*^*2*^ on different incident angles.

According to above simulation analyses, a group of optimized structural parameters are confirmed finally, i.e. *H* = 500 μm, *L* = 15 μm, *W*_0_ = 100 μm and *P* = 400 μm. On the basis of the optimized structure, we studied the influence of different incident angles (*α*) on the normalized *E*^2^ in the place of the detector as shown in Fig. 6.

The *E*^2^ amplitude gradually increased when changing *α* from −90° to 0°, the maximum normalized *E*^2^ is 1 when *α* = 0°. And when increasing *α* from 0° to 90°, the *E*^2^ amplitude gradually decreased as shown in Fig. 6. Due to the nonzero electric field component in case of oblique incidence, which propagates along the X-axis, unable to be diffracted or reflected by the reflective grating-coupled structure. So, it makes the *E*^2^ amplitude in case of oblique incidence (*α*≠0) smaller than that case of normal incidence (*α* = 0).

## Methods

### Reflective grating-coupled structure

In the design of THz array detector chip, Different kinds of structures are usually added on the substrate to enhance the detection efficiency. A metallic layer is deposited on the back of the substrate to make up a metallic Fabry-Perot (F-P) cavity. The incident signal can be enhanced on the substrate surface if the substrate thickness (i.e. F-P cavity) and the wavelength satisfy Eq. (1):

1 $$2nH=({2k}_{1}-\,1)\frac{\lambda }{2},({k}_{1}=1,2,3,\cdot \cdot \cdot ).$$

where *n* and *H* are the refractive index (*n* = 3.45 for Si) and the substrate thickness respectively.

For this current reflective grating-coupled structure, the incident signal could be focused on the substrate surface highly effectively by the reflection and diffraction effects. The transmission signals at the grating gear place can be totally reflected, which is similar to the case of F-P cavity. Besides, the transmission signals at the groove place can be diffracted due to the grating effect. Therefore, the reflected and diffracted signals in substrate can be effectively coupled to the substrate surface, and the signals in the place of the detector can be enhanced by the interference effect. Therefore, except for the Eq. (1), the other two equations should be considered as:

2 $$2nH={k}_{2}\cdot \lambda +nd(1+\,\sec \,\beta ),({k}_{2}=1,2,3,\cdot \cdot \cdot ).$$

And

3 $$m\lambda =nP(\sin \,\alpha +\,\sin \,\beta ),(m=1,2,3,\cdot \cdot \cdot ).$$

where *m* is the diffraction order, *n* is the refractive index (*n* = 3.45 for Si), *H* is the substrate thickness, *P* is the grating period, *α* and *β* are the incident angle and diffraction angle respectively. For a plane wave, when *α* = 0, Eq. (3) can be rewritten as

4 $$m\lambda =nP\,\sin \,\beta ,(m=1,2,3,\cdot \cdot \cdot ).$$

The zero order diffraction corresponds to *m* = 0 in the Eq. (4). At this time, the diffraction angle is independent of the wavelength and the signal cannot be diffracted by the grating. When *m*≠0, the diffraction angle varies with the wavelength.

### Fabrication process

The reflective grating-coupled structure and the F-P resonator were respectively applied in the preparation of the Nb_5_N_6_ detector chip. Firstly, 110 nm-thick Nb_5_N_6_ film and 220 nm-thick Au electrodes were deposited on Si substrates with different thickness by using radio frequency (RF) magnetron sputtering. Then, the fabrication of Nb_5_N_6_ microbridge was finished after the steps of photolithography and reactive ion etching (RIE)^9^. The size of the single Nb_5_N_6_ detector was 210 μm × 185 μm, and the period of the array detectors was 320 μm as shown in Fig. 3(b). Secondly, the fabrication of the F-P resonator and the reflective grating-coupled structure on the back of Si substrates. A 220 nm gold layer was deposited on the back of substrate to form the F-P resonator. The reflective grating-coupled structure was formed on the back of the substrate through the photolithography and the inductively coupled plasma (ICP) technology. In ICP process, RF and ICP power were 30 W and 1000 W respectively. The 15 minutes ICP etching took place in a gas mixture of SF_6_ (50 sccm) and C_4_F_8_ (50 sccm) at a total pressure of 20 mTorr. The groove depth about 15 μm was formed. Finally, a 220 nm gold layer was deposited on the top. The fabricated grating-coupled structure is shown in Fig. 3(a).

### Measurement setup

**Figure 7:**
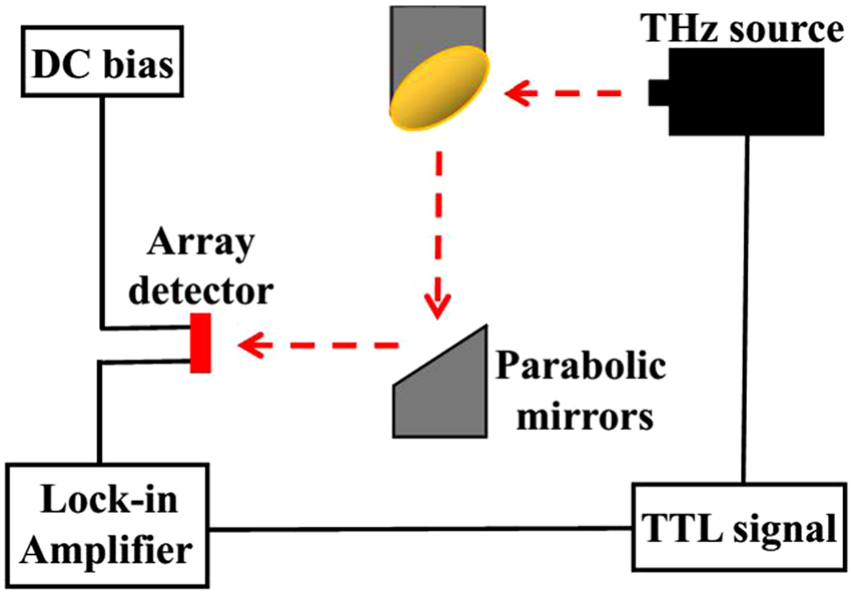
Schematic of the test system constructed using two off-axis parabolic mirrors.

A quasi-optical test system constructed using two off-axis parabolic mirrors is shown in Fig. 7. The incident signal from a signal source, whose frequency can be tuned from 0.26 THz to 0.365 THz, was modulated using a 1 KHz TTL signal. The input power was measured by a thermal power sensor (OPHIR, 3 A-P-THz) at Nb_5_N_6_ array detector place. The voltage responses of the Nb_5_N_6_ detector which was biased at 0.4 mA were read out by a lock-in amplifier^21^.
